# Bayesian evaluation of effect size after replicating an original study

**DOI:** 10.1371/journal.pone.0175302

**Published:** 2017-04-07

**Authors:** Robbie C. M. van Aert, Marcel A. L. M. van Assen

**Affiliations:** 1 Department of Methodology and Statistics, Tilburg University, the Netherlands; 2 Department of Sociology, Utrecht University, the Netherlands; Ghent University, BELGIUM

## Abstract

The vast majority of published results in the literature is statistically significant, which raises concerns about their reliability. The Reproducibility Project Psychology (RPP) and Experimental Economics Replication Project (EE-RP) both replicated a large number of published studies in psychology and economics. The original study and replication were statistically significant in 36.1% in RPP and 68.8% in EE-RP suggesting many null effects among the replicated studies. However, evidence in favor of the null hypothesis cannot be examined with null hypothesis significance testing. We developed a Bayesian meta-analysis method called *snapshot hybrid* that is easy to use and understand and quantifies the amount of evidence in favor of a zero, small, medium and large effect. The method computes posterior model probabilities for a zero, small, medium, and large effect and adjusts for publication bias by taking into account that the original study is statistically significant. We first analytically approximate the methods performance, and demonstrate the necessity to control for the original study’s significance to enable the accumulation of evidence for a true zero effect. Then we applied the method to the data of RPP and EE-RP, showing that the underlying effect sizes of the included studies in EE-RP are generally larger than in RPP, but that the sample sizes of especially the included studies in RPP are often too small to draw definite conclusions about the true effect size. We also illustrate how *snapshot hybrid* can be used to determine the required sample size of the replication akin to power analysis in null hypothesis significance testing and present an easy to use web application (https://rvanaert.shinyapps.io/snapshot/) and R code for applying the method.

## Introduction

Most findings published in the literature are statistically significant [1–3] and are subsequently interpreted as nonzero findings. However, when replicating these original published studies in conditions as similar as possible to the original studies (so-called direct replications), replications generally provide lower estimates of the effect size that often are not statistically significant and are interpreted as suggesting a null effect. For instance, in medicine findings of only 6 out of 53 (11.3%) landmark studies on the field of hematology and oncology were confirmed in replication studies [4]. In psychology, the Reproducibility Project Psychology (RPP; [5]) replicated 100 studies published in major journals in 2008. Of the 97 original findings reported as statistically significant, only 35 (36.1%) had a statistically significant effect in the replication, and 81 of 97 (83.5%) findings were stronger in the original study. In economics, the Experimental Economics Replication Project (EE-RP; [6]) replicated 18 studies published in high-impact journals. Of 16 findings that were statistically significant in the original study, 11 (68.8%) were statistically significant in the replication, and 13 of 16 (81.3%) had a stronger effect in the original study.

Interpreting the results of the replicability projects as providing evidence of many true null effects among the originally published studies has received criticism (e.g., [7, 8]). For instance, Maxwell, Lau, and Howard [8] argue that, although the replication in RPP generally had higher statistical power than the original study, the power of the replication was still too low to consider as evidence in favor of the null hypothesis. Consequently, the statistically nonsignificant findings of many replications are also consistent with a true nonzero, albeit small effect.

Many researchers adhere to null hypothesis significance testing (NHST) when evaluating the results of replications, and conclude based on a nonsignificant replication that the original study does not replicate [9]. However, such a vote counting procedure has been largely criticized in the context of a meta-analysis (e.g., [10], Chapter 28) and comes along with three fundamental problems. First, one cannot obtain evidence in favor of the null hypothesis of a true zero effect with NHST (e.g., [11]). Second, NHST does not tell us the size of the effect. Third, not all available information about the underlying effect is used in NHST because evidence obtained in the original study is ignored. What we need are methods providing evidence on the common true effect underlying both the original study and replications.

The method that immediately comes to mind when the goal is to estimate effect size based on several studies is meta-analysis. Two different traditional meta-analytic models can generally be distinguished: fixed-effect and random-effects model. Fixed-effect meta-analysis assumes that one common true effect underlies all observed effect sizes, whereas random-effects meta-analysis assumes observed effect sizes arise from a (normal) distribution of true effect sizes [12]. When one study is a direct replication of another, fixed-effect rather than random-effects meta-analysis seems to be the most appropriate method because the two studies are very similar. A small amount of heterogeneity in true effect size, however, may be possible since there could be minor discrepancies in for instance the studied population or experimental design as was sometimes the case in RPP. Publication bias is universally recognized as a major threat to the validity of meta-analyses, leading to overestimation of effect size (e.g., [13–15]). Publication bias is the suppression of statistically nonsignificant results from being published [16]. Evidence of publication bias and as a consequence overestimation of effect size is omnipresent (e.g., [1–3, 14]), and is also obvious from the aforementioned results of the replicability projects; almost all original findings were statistically significant whereas the replication findings were not, and the large majority of original effect size estimates was larger than those in the replication. Hence, traditional meta-analysis will be biased as well and will not suffice. A meta-analysis method is needed that takes into account the statistical significance of the original study, thereby adjusting for publication bias.

The present paper develops and applies a Bayesian meta-analytic method, called *snapshot hybrid*, to evaluate the effect size underlying an original study and replication. A requirement for applying the method is that the effect size of the original study is statistically significant. This requirement hardly restricts the applicability of the proposed method since the vast majority of published studies contain statistically significant results [1–3] and replications are often conducted when statistical significance is observed.

The snapshot hybrid has many desirable properties. First, the method has few assumptions. It assumes both studies estimate the same true effect size and the effect size in the original study and replication is normally distributed. The second desirable property is that, as opposed to fixed-effect meta-analysis, our method adjusts for publication bias when evaluating the underlying true effect size by taking into account statistical significance of the original study. Third, it provides a very simple interpretation of the magnitude of the true effect size. Its main output is the posterior model probability of a zero, small, medium, and large effect (i.e., probability of a model after updating the prior model probability with the likelihood of the data). Consequently, as opposed to NHST, it also quantifies the evidence in favor of the null hypothesis, relative to a small, medium, and large hypothesized effect. One high posterior model probability suggests certainty about the magnitude of the true effect, whereas several substantial nonzero posterior model probabilities indicate that the magnitude of the effect is rather uncertain. Fourth, the method has great flexibility in dealing with different prior information. Although the method’s default prior model probabilities are equal (i.e., zero, small, medium and large effect are equally likely), using a simple formula one can recalculate the posterior model probabilities for other prior model probabilities, without having to run the analysis again.

The goal of the present paper is fourfold. First, we explain snapshot hybrid and examine its statistical properties. Second, we apply the method to the data of RPP and EE-RP to examine evidence in favor of zero, small, medium, and large true effects. Particularly, we verify if interpreting the statistically nonsignificant findings of replication studies in psychology as evidence for null effects is appropriate. Third, we describe, analogous to conducting a power analysis for determining the sample size in a frequentist framework, how the proposed method can be used to compute the required sample size for the replication in order to get a predefined posterior model probability of the true effect size being zero, small, medium, or large. This goal acknowledges that our method is not only relevant for evaluating and interpreting replicability of effects. Replicating other’s research is often the starting point for new research, where the replication is the first study of a multi-study paper [17]. Fourth, we present a web application and R code allowing users to evaluate the common effect size of an original study and replication using snapshot hybrid.

The next section provides a hypothetical example of an original study and replication by Maxwell et al. [8], and illustrates the problem of evaluating the studies’ underlying true effect size. The subsequent section explains snapshot hybrid, and is illustrated by applying it to the example of Maxwell et al. [8]. Then, the statistical properties of our method are examined analytically. Subsequently, the method was applied to the results of RPP and EE-RP. How the required sample size of the replication can be determined with the proposed method in order to achieve a predefined posterior model probability for a hypothesized effect size (zero, small, medium, or large) is discussed next. Then, the computer program is described to determine this required sample size, followed by a conclusion and discussion section.

## Methods related to snapshot hybrid

The proposed snapshot hybrid method is related to several other methods. The meta-analysis methods *p*-uniform [15, 18] and *p*-curve [19] also take statistical significance of studies’ effect sizes into account in order to correct the meta-analytic effect size estimate for publication bias. The effect size estimate of *p*-uniform and *p*-curve is equal to the effect size where the statistically significant *p*-values conditional on being statistically significant are uniformly distributed. Both methods have been shown to provide accurate estimates of the underlying true effect size in case of publication bias, but only if the amount of heterogeneity in studies’ true effect size is modest [15, 18–20]. We also wrote a paper where we use frequentist statistics to evaluate the common effect size underlying an original study and a replication, taking into account the statistical significance of the original study [21]. This method estimates effect size, provides a confidence interval, and enables testing of the common effect. Advantages of the Bayesian method presented here are that interpretation of its results is more straightforward, and evidence in favor of the null hypothesis is quantified.

Another related paper is a Bayesian re-analysis of the results of RPP [22]. In this paper, Bayes factors were computed for each original study and replication separately, comparing the null hypothesis of no effect with an alternative hypothesis suggesting that the effect is nonzero. For the original studies, publication bias was taken into account when computing Bayes factors by using Bayesian model averaging over four different publication bias models. The most important differences of our Bayesian method and their re-analyses, which we interpret as advantages of our methodology, are: (i) they do not evaluate the underlying effect size, but test hypotheses for original study and replication separately, (ii) they make strong(er) assumptions on publication bias, using Bayesian model averaging over four different models of publication bias, (iii) their methodology lacks flexibility with dealing with different prior information (i.e. another prior requires rerunning the analysis), and (iv) they did not provide software to run the analysis. Both Etz and Vandekerckhove [22] and van Aert and van Assen [21] conclude that for many RPP findings no strong conclusions can be drawn on the magnitude of the underlying true effect size.

## Example by Maxwell, Lau, and Howard [8]

We will illustrate snapshot hybrid using a hypothetical example provided by Maxwell et al. [8] with a statistically significant original study and nonsignificant replication. They use their example to illustrate that so-called failures to replicate in psychology may be the result of low statistical power in single replication studies. This example was selected because it reflects an often occurring situation in practice. For instance, 62% of the 100 replicated studies in RPP [5] and 39% of the 18 replicated studies in EE-RP [6] did not have a statistically significant effect, as opposed to the effect in the original study. Hence, researchers often face the question what to conclude with respect to the magnitude of the true effect size based on a statistically significant original effect and a nonsignificant replication effect. Does an effect exist? And if an effect exists, how large is it?

The example employs a balanced two-independent groups design. The original study, with 40 participants per group, resulted in Cohen’s *d* = 0.5 and *t*(78) = 2.24 (two-tailed *p*-value = .028), which is a statistically significant effect if tested with α = .05. A power analysis was used by Maxwell et al. [8] to determine the required sample size in the replication to achieve a statistical power of .9 using a two-tailed test, with an expected effect size equal to the effect size observed in the original study. The power analysis revealed that 86 participants per group were required. The observed effect size in the replication was Cohen’s *d* = 0.23 with *t*(170) = 1.50 (two-tailed *p*-value = .135), which is not statistically significant if tested with α = .05.

In our analyses, like in RPP and EE-RP, we transform effect sizes to correlation coefficients. Correlation coefficients are bounded between -1 and 1, and easy to interpret. Transforming original and replication effect sizes to correlations using $r_{o}=\frac{d}{\sqrt{d+4}}$ (e.g., [10], p. 48) yields *r*_*o*_ = 0.243 and *r*_*r*_ = 0.114 for original and replication effect size, respectively. Testing individual correlations as well as combining correlations in a meta-analysis is often done using Fisher-transformed correlation coefficients [10] (Chapter 6), since these follow a normal distribution with variance 1/(*N* − 3) with *N* being the total sample size [23]. The Fisher-transformed correlations (*θ*) are ${\hat{\theta }}_{o}=0.247$ and ${\hat{\theta }}_{r}=\;0.115$ with standard errors .114 and .0769, respectively. Statistically combining the two effects by means of fixed-effect meta-analysis yields $\hat{\theta }=0.156$ with standard error 0.0638, which is statistically significant (two-tailed *p* = .0142), suggesting a positive effect. Transforming the results of the meta-analysis to correlation coefficients yields the effect size estimate of 0.155 (95% confidence interval; 0.031 to 0.274). Although fixed-effect meta-analysis suggests a positive effect size, it should be interpreted with caution because of the generally overestimated effect size in the original study due to publication bias.

## Snapshot Bayesian hybrid meta-analysis

The snapshot Bayesian hybrid meta-analysis method, *snapshot hybrid* for short, is a *meta-analysis* method because it combines both the original and replication effect size to evaluate the common true effect size. It is a *hybrid* method because it only takes the statistical significance of the original study into account, whereas it considers evidence of the replication study as unbiased. The method is *Bayesian* because it yields posterior model probabilities of the common true effect size. Finally, it is called *snapshot* because only four snapshots or slices of the posterior distribution of effect size are considered, i.e. snapshots/slices at hypothesized effect sizes equal to zero (ρ = 0), and small (ρ = 0.1), medium (ρ = 0.3), and large (ρ = 0.5) correlations [24] (Chapter 4). We selected these four hypothesized effect sizes, because applied researchers are used to this categorization of effect size. Moreover, point hypotheses enable recalculating the posterior model probabilities for other than uniform encompassing prior distributions (i.e., prior model probabilities derived from other prior distributions than a uniform distribution that results in equal probabilities for the hypothesized effect sizes) as we will show later.

Two assumptions are underlying snapshot hybrid. First, the same effect (i.e., fixed effect) has to be underlying the original study and replication. This assumption seems to be reasonable if the replication is exact although small amounts of heterogeneity may arise if there are minor discrepancies in studied population or experimental design. Exact replications are often conducted as the first study of a multi-study paper [17]. Second, effect size in the original study and replication are assumed to be normally distributed, which is a common assumption in meta-analysis [25]. Furthermore, the original study is required to be statistically significant. This requirement hardly restricts the range of application of the method because most studies in the social sciences contain statistically significant results, particularly in psychology with percentages of about 95% (e.g., [1, 3]) or even 97%, as in the RPP [5] and also 89% in the EE-RP. Note that, even if publication bias was absent in science, snapshot hybrid *should* be used if a researcher chooses to replicate an original study because of its statistical significance. It is precisely this selection that biases methods that do not correct for statistical significance, similar to how selecting only ill people for treatment or high scoring individuals on an aptitude test results in regression to the mean when re-tested.

The snapshot hybrid consists of three steps. First, the likelihood of the effect sizes of the original study and replication is calculated conditional on four hypothesized effect sizes (zero, small, medium, and large). Second, the posterior model probabilities of these four effect sizes are calculated using the likelihoods of step 1 and assuming equal prior model probabilities. Equal prior model probabilities are selected by default, because this refers to an uninformative prior distribution for the encompassing model. Third, when desired, the posterior model probabilities can be recalculated for other than equal prior model probabilities. We will explain and illustrate each step by applying the method to the example of Maxwell et al. [8].

In the first step, the combined likelihood of the effect size of the original study (${\hat{\theta }}_{o}$) and replication (${\hat{\theta }}_{r}$) for each hypothesized effect size (*θ*) is obtained by multiplying the densities of the observed effect sizes:

$$L(\theta )=f({\hat{\theta }}_{o},{\hat{\theta }}_{r}\left|\theta )=f_{o}({\hat{\theta }}_{o}\right|\theta )\times f_{r}({\hat{\theta }}_{r}|\theta )$$ (1)

**Fig 1:**
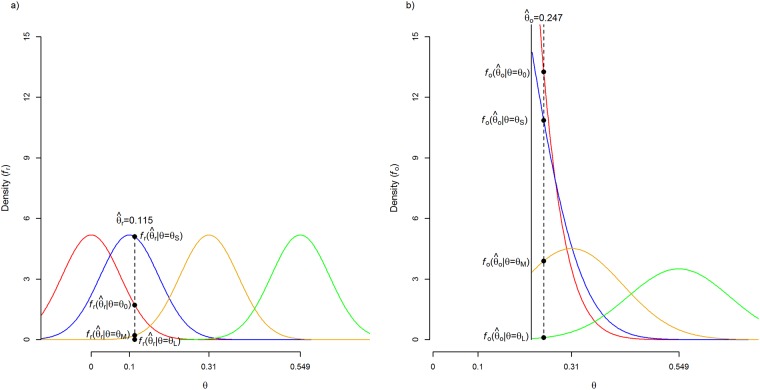
Probability density functions of the replication (panel a) and transformed original effect size when statistical significance is taken into account (panel b). The four hypothesized effect sizes (zero, small, medium, and large) are denoted by θ = 0 (0, red distribution), θ = 0.1 (S, blue distribution), θ = 0.31 (M, yellow distribution), and θ = 0.549 (L, green distribution). The dashed vertical line refers to the observed effect sizes in the hypothetical example of Maxwell et al. [8] for the replication (panel a) and original study (panel b). The dots on the vertical dashed line refer to densities for no, small, medium, and large effect in the population.

Note that densities and likelihood are based on Fisher-transformed correlations and hypothesized effect sizes. Fig 1a shows the probability density functions and densities of the observed effect size of the replication (${\hat{\theta }}_{r}=\;.115$). The four density functions follow a normal distribution with means *θ*_*0*_ = 0 (red distribution), *θ*_*S*_ = 0.1 (blue distribution), *θ*_*M*_ = 0.31 (yellow distribution), *θ*_*L*_ = 0.549 (green distribution), and standard deviation ${\hat{\sigma }}_{r}=\;.0769$. The densities or heights at ${\hat{\theta }}_{r}=\;.115$ (see vertical dashed line) are 1.705, 5.096, 0.210, 0, for a zero, small, medium, large true effect, respectively.

Fig 1b shows the four density functions and densities of the observed effect size in the original study (${\hat{\theta }}_{o}=.247$). Colors red, blue, yellow, and green refer again to distributions of a zero (θ_0_), small (θ_S_), medium (θ_M_), and large (θ_L_) hypothesized effect, respectively. The density functions take statistical significance of the original finding into account by computing the density of ${\hat{\theta }}_{o}$ conditional on the study being statistically significant, i.e. by *truncating* the densities at the critical value of the Fisher-transformed correlation (*θ*_*cv*_). A two-tailed test with *α* = .05 is assumed reflecting common practice in social science research where two-tailed tests are conducted and only the results in one direction get published. The truncated densities are calculated as

$$f_{o}\left({\hat{\theta }}_{o}|\theta \right)=\frac{\phi \left(\frac{{\hat{\theta }}_{o}-\theta }{{\hat{\sigma }}_{o}}\right)}{1-\Phi \left(\frac{\theta_{cv}-\theta }{{\hat{\sigma }}_{o}}\right)}$$ (2)

with *ϕ* and Φ being the standard normal density and cumulative distribution function, respectively. The denominator of (2) also represents the power of the test of no effect if the hypothesized effect size is equal to *θ*. Note how the conditional density *f*_*o*_ in Fig 1b of a just significant correlation increases when the hypothesized correlation decreases; the conditional density *f*_*o*_ is virtually identical to the unconditional density for large hypothesized effect size (because statistical power is close to 1), whereas the conditional density is 40 times larger (i.e., 1/(α/2)) than the unconditional density function for *θ* = 0. The densities at ${\hat{\theta }}_{o}=.247$ are 13.252, 10.852, 3.894, 0.105 for a zero, small, medium, large hypothesized effect, respectively. Note that after taking the statistical significance of the original finding into account, the density is highest for *θ*_*0*_ and *θ*_*S*_ and substantially lower for *θ*_*M*_, and *θ*_*L*_. Hence, it is less likely that ${\hat{\theta }}_{o}$ stems from a population with a medium or large effect size than from a population with no effect or a small effect size. The first row of Table 1 presents the likelihoods of the observed effect sizes as a function of hypothesized effect size, after multiplying the studies’ densities with Eq (1). The likelihood is largest for a small hypothesized effect in comparison with no, medium, and large hypothesized effect, suggesting that there is most probably a small true effect underlying the original study and replication.

**Table 1:** Likelihoods and posterior model probabilities for zero, small, medium, and large hypothesized correlations for the example of Maxwell et al. [8].

	Prior	Method	Zero (ρ_S_ = 0)	Small (ρ_S_ = 0.1)	Medium (ρ_S_ = 0.3)	Large (ρ_S_ = 0.5)
Likelihood			22.594	55.304	0.819	0
Posterior model probabilities	Uniform	Snapshot hybrid	.287	.703	.010	0
		Snapshot naïve	.063	.866	.071	0
	*p*_*0*_ = 2	Snapshot hybrid	.446	.546	.008	0
	*p*_*0*_ = 6	Snapshot hybrid	.707	.289	.004	0
	*N*(0,1)	Snapshot hybrid	.288	.702	.010	0

Posterior model probabilities of one snapshot (ρ_S_) relative to the others are calculated with the snapshot hybrid (second row and last three rows) and without correcting for statistical significance (snapshot naïve, third row). For snapshot hybrid, posterior model probabilities are calculated for four different sets of prior model probabilities; equal prior model probabilities (i.e., uniform encompassing model), prior model probabilities where the hypothesized zero effect gets a weight (*p*_*0*_) 2 or 6 times higher than the other hypothesized effects, and prior model probabilities when a normal distribution with mean and variance equal to 0 and 1 is the encompassing model, respectively.

In the second step, the posterior model probability π_x_ of each model with hypothesized effect size *x* is calculated using

$$\pi_{x}=\frac{L(\theta =x)}{L(\theta =\theta_{0})+L(\theta =\theta_{S})+L(\theta =\theta_{M})+L(\theta =\theta_{L})}$$ (3)

where *x* refers to either a zero (θ_0_), small (θ_S_), medium (θ_M_), or large (θ_L_) hypothesized effect size. This posterior model probability is a relative probability because it quantifies the amount of evidence for a model with a particular hypothesized effect size relative to the other included models. Since all likelihoods are weighed equally, implicitly equal prior model probabilities are assumed in Eq (3). The second row of Table 1 (method ‘snapshot hybrid’, uniform prior) presents the four posterior model probabilities of snapshot hybrid for the example. The posterior model probabilities indicate that after observing correlations *r*_*o*_ = 0.243 and *r*_*r*_ = 0.114, the evidence in favor of the null hypothesis slightly increased from .25 to .287, increased a lot (from .25 to .703) in favor of a small hypothesized effect size, and decreased a lot for a medium and large hypothesized effect size.

For the sake of comparison, we also calculated the posterior model probabilities using a method we call *snapshot naïve* because it incorrectly does not take the statistical significance of the original finding into account (i.e., without truncating the density at *θ*_*cv*_). Its results are presented in the third row of Table 1 (method ‘snapshot naïve’, uniform encompassing prior distribution). These uncorrected posterior model probabilities provide stronger evidence in favor of a small effect relative to a zero, medium, and large effect, although posterior model probabilities for both a zero and medium hypothesized effect are still larger than zero (.06). Comparing the results of applying snapshot hybrid to those of snapshot naïve to the example shows that snapshot hybrid assigns larger posterior model probabilities to zero hypothesized effect size than snapshot naïve. This always holds. More generally, the snapshot naïve first-order stochastically dominates snapshot hybrid, i.e., snapshot naïve’s cumulative posterior model probabilities exceed those of snapshot hybrid. The evaluation of snapshot hybrid may even suggest that the true effect size is smaller than the estimates of *both* the original study and replication. The latter typically occurs when the original effect size is just statistically significant (i.e., has a *p*-value just below .05) and the replication effect size has the same sign as the original effect.

Finally, in the third step the posterior model probabilities of a hypothesized effect size relative to the other hypothesized effect sizes may be recalculated using other than equal model probabilities. The posterior model probability $\pi_{x}^{*}$ for hypothesized effect size *x* can be recalculated using

$$\pi_{x}^{*}=\frac{p_{x}\pi_{x}}{p_{0}\pi_{0}+p_{S}\pi_{S}+p_{M}\pi_{M}+p_{L}\pi_{L}},$$ (4)

with prior model probabilities or weights *p*, and posterior model probabilities π calculated with Eq (3) assuming equal uniform prior probabilities. Note that $\pi_{x}^{*}=\pi_{x}$ for equal prior model probabilities. The values of *p*_*x*_, with *x* referring to no (0), small (S), medium (M), or large hypothesized effect (L), can simply be derived from the prior density function of the researcher.

Simple and conservative prior model probabilities are to assign, for instance, a two or even six times higher prior model probability to a zero hypothesized effect than to any of the other hypothesized effects. Note that other prior model probabilities can also be used, and that these probabilities can also be specified for other hypothesized effect sizes than zero. Substituting *p*_*0*_ = 2 and *p*_*0*_ = 6 (and *p*_*S*_, *p*_*M*_, and *p*_*L*_ all equal to 1) and the posterior model probabilities presented in row “uniform snapshot hybrid” of Table 1, yields the recalculated posterior model probabilities presented in the two subsequent rows of Table 1. Naturally, more conservative prior model probabilities yield stronger evidence in favor of a hypothesized zero effect, with posterior model probabilities increasing from .287 (uniform prior) to .707 (*p*_*0*_ = 6).

The posterior model probabilities can also be recalculated when a continuous prior is specified for the encompassing model, for instance a normal distribution with mean and variance equal to 0 and 1, respectively, denoted by *N*(0,1) in Table 1. This normal prior yields prior model probabilities at *θ* = 0, *θ* = 0.1, *θ* = 0.31, *θ* = 0.549 of *p*_*0*_ = 0.263, *p*_*S*_ = 0.261, *p*_*M*_ = 0.250, *p*_*L*_ = 0.226, which are close to the equal prior model probabilities. This yields the recalculated posterior model probabilities in the last row of Table 1, again showing that assigning higher prior model probability to a hypothesized zero effect results in stronger evidence in favor of the null hypothesis. To sum up, the posterior model probabilities can be recalculated without doing the Bayesian analysis again, by applying Eq (4) using other prior model probabilities. Second, the example demonstrates that the prior model probabilities can have substantial effects on the posterior model probabilities, particularly if there is no (very) strong evidence for a hypothesized effect size.

## Analytical evaluation of statistical properties

We evaluated the statistical properties of snapshot hybrid by comparing it to snapshot naïve. This comparison demonstrates that effect size evaluation often suggests larger effect sizes than the true effect size if statistical significance of the original study is not taken into account. Statistical properties of the methods were evaluated with the correlation coefficient as the effect size measure of interest. However, both methods can also be applied to other effect size measures (e.g., standardized mean differences).

### Method

We analytically approximated the statistical properties of both snapshot hybrid and snapshot naïve using numerical integration of the joint probability density function (pdf) of the statistical significant original effect size and effect size in the replication. This joint pdf is a function of the true effect size and both effect sizes’ standard error. The joint pdf of the statistically significant observed original effect size and the effect size of the replication was approximated by creating an equally spaced grid of 5,000 x 5,000 values. The pdf of the statistically significant observed original effect sizes was approximated by first selecting 5,000 equally spaced cumulative probabilities given that the effects sizes that accompanied these probabilities were statistically significant. A one-tailed Fisher-*z* test with α = .025 was used to determine the critical value for observing a statistically significant effect size in the original study because this corresponds to a common practice in social science research where two-tailed hypothesis tests are conducted and only the results in the predicted direction are reported. For instance, under the null hypothesis this means that the cumulative probabilities range from $1-0.025+\frac{\left(1\times .025\right)}{5,001}=.975005$ to $1-0.025+\frac{\left(5,000\times .025\right)}{5,001}=.999995$. All these cumulative probabilities were then transformed to Fisher-transformed correlation coefficients given a true effect size and standard error to approximate the pdf of the original effect size. The pdf of the replication’s observed effect size given a true effect size and standard error was created in a similar way as the pdf of the original study’s observed effect size, but there was no requirement for the effect size in the replication to be statistically significant. Hence, 5,000 equally spaced cumulative probabilities ranging from $\frac{1}{5,001}=.00019996$ to $\frac{5,000}{5,001}=.9998$ were selected and the pdf of the observed effect size in the replication was obtained by transforming these probabilities to Fisher-transformed correlation coefficients. Combining the marginal pdfs of the observed effect size in the original study and replication resulted in an approximation of the joint pdf consisting of 25,000,000 different combinations of effect sizes that was used for evaluating the statistical properties of snapshot hybrid and snapshot naïve. Both methods were applied to each combination of effect size in the original study and replication.

In order to examine the performance of the methods under different conditions, joint pdfs were created by varying two factors: total sample size for the original study and replication (*N*) and true effect size (ρ). Six different sample sizes were selected (*N* = 31; 55; 96; 300; 1,000; 10,000) and were imposed to be equal in the original study and replication. Sample sizes of 31, 55, and 96 refer to the first quartile, medium, and third quartile of the observed sample sizes of the original study in RPP [5]. Larger sample sizes were also included for two reasons. First, large sample sizes enable us to examine large sample properties of our method, such as convergence of the methods to the correct hypothesized effect size. Second, bias of snapshot naïve can be examined with large sample sizes, because bias is expected to disappear for very large sample size whenever true effect size exceeds zero. For the true effect size, we selected ρ = 0 (no effect), 0.1 (small effect), 0.3 (medium effect), 0.5 (large effect), which correspond to the methods’ snapshots or hypothesized effect sizes. Our analysis used equal prior model probabilities, assigning probabilities of .25 to each hypothesized effect size.

Posterior model probabilities of snapshot hybrid and snapshot naïve at the four hypothesized effect sizes were computed using Eq (3) for each of the 25,000,000 different combinations of effect sizes. Performances of both methods was then evaluated with respect to three outcomes. The first outcome was the expected value of the posterior model probability for each hypothesized effect size. Second, we calculated the proportion that the posterior model probability of a particular hypothesized effect size relative to the other hypothesized effect sizes was larger than .25, which amounts to the probability that evidence in favor of the true hypothesis increases after observing the data. The third outcome was the proportion that the posterior model probability of a particular hypothesized effect size relative to the other hypothesized effect sizes was larger than .75. This proportion corresponds to a Bayes Factor of 3 when comparing a particular hypothesized effect size to the other hypothesized effect sizes. Since a Bayes Factor exceeding 3 is interpreted as *positive* evidence (e.g., [26]), we interpret posterior model probabilities of .75 or more as positive evidence in favor of that hypothesized effect size. Note that selecting a posterior model probability of 0.75 (and Bayes Factor of 3) is a subjective choice, and that selecting other posterior model probabilities (and Bayes Factors) for the analyses was also possible. In our analyses, we expect all three outcomes to increase in sample size for snapshot hybrid, but not always for the biased snapshot naïve method.

Computations were conducted in the statistical software R and the parallel package was used for parallelizing the computations [27]. Computer code for the computations is available at https://osf.io/xrn8k/.

### Results on statistical properties

#### Expected value of the posterior model probability

Table 2 presents the expected values of the posterior model probabilities of snapshot hybrid and snapshot naïve for four different snapshots (ρ_S_). The posterior model probabilities are presented for different sample sizes (*N*) and true effect sizes (ρ). Results for sample sizes per group equal to 10,000 are not shown since expected posterior model probabilities of both methods are always equal to 1 for the correct snapshot and to 0 for the incorrect snapshot. The bold values in the columns for snapshot hybrid and snapshot naïve indicate the posterior model probability for that particular snapshot that matches the true effect size. Hence, the bold values in these columns should be higher than the posterior model probabilities for the other snapshots. The final column shows the expected value of the estimate of traditional fixed-effect meta-analysis.

**Table 2:** Expected values of the posterior model probabilities of the snapshot hybrid, snapshot naïve, and traditional fixed-effect meta-analysis (FE).

		Snapshot Hybrid				Snapshot Naïve				FE
	*N*	ρ_S_ = 0	ρ_S_ = 0.1	ρ_S_ = 0.3	ρ_S_ = 0.5	ρ_S_ = 0	ρ_S_ = 0.1	ρ_S_ = 0.3	ρ_S_ = 0.5	
ρ = 0	31	**0.466**	0.36	0.151	0.023	**0.177**	0.336	0.411	0.076	0.215
	55	**0.535**	0.375	0.089	0.002	**0.212**	0.479	0.304	0.005	0.16
	96	**0.601**	0.368	0.03	0	**0.241**	0.648	0.112	0	0.12
	300	**0.757**	0.243	0	0	**0.338**	0.662	0	0	0.068
	1,000	**0.948**	0.052	0	0	**0.758**	0.242	0	0	0.037
ρ = 0.1	31	0.36	**0.351**	0.231	0.057	0.11	**0.258**	0.485	0.147	0.268
	55	0.375	**0.403**	0.211	0.011	0.111	**0.367**	0.501	0.021	0.215
	96	0.368	**0.481**	0.15	0	0.101	**0.552**	0.347	0	0.177
	300	0.243	**0.745**	0.012	0	0.04	**0.94**	0.02	0	0.127
	1,000	0.052	**0.948**	0	0	0.007	**0.993**	0	0	0.103
ρ = 0.3	31	0.151	0.231	**0.367**	0.25	0.027	0.099	**0.456**	0.417	0.381
	55	0.089	0.211	**0.523**	0.178	0.012	0.089	**0.663**	0.236	0.337
	96	0.03	0.15	**0.738**	0.082	0.003	0.066	**0.842**	0.089	0.312
	300	0	0.012	**0.985**	0.003	0	0.008	**0.989**	0.003	0.3
	1,000	0	0	**1**	0	0	0	**1**	0	0.3
ρ = 0.5	31	0.023	0.058	0.25	**0.669**	0.002	0.014	0.188	**0.796**	0.516
	55	0.002	0.011	0.178	**0.808**	0	0.002	0.146	**0.851**	0.499
	96	0	0	0.082	**0.918**	0	0	0.075	**0.925**	0.498
	300	0	0	0.003	**0.997**	0	0	0.003	**0.997**	0.499
	1,000	0	0	0	**1**	0	0	0	**1**	0.5

ρ denotes the effect size in the population, *N* is the sample size in the original study and replication, and ρ_S_ refers to the snapshots of effect size.

Expected values of the posterior model probabilities of snapshot hybrid at the correct snapshot (e.g., ρ_S_ = 0 if ρ = 0 and ρ_S_ = 0.1 if ρ = 0.1) increase as the sample size increases, as they should (bold values in first four columns in Table 2). Expected values of the posterior model probabilities are close to .75 for ρ = 0, ρ = 0.1, and ρ = 0.3 at *n*_i_ = 300, and at *N* = 55 for ρ = 0.5. Snapshot hybrid has difficulties distinguishing whether an effect is absent (ρ = 0) or small (ρ = 0.1) for *N* < 1,000 because the expected values of the posterior model probabilities at ρ_S_ = 0 and ρ_S_ = 0.1 are close to each other for both effect sizes. Even if *N* = 1,000, the expected value of the posterior model probability of ρ = 0 at snapshot ρ_S_ = 0.1 is .052 and the same holds for the expected value of the posterior model probability of ρ = 0.1 at snapshot ρ_S_ = 0.

The expected values of the posterior model probability of snapshot naïve also increase as the sample size increases (bold values in columns seven to ten in Table 2). However, the performance of snapshot naïve is worse than of snapshot hybrid for ρ = 0 for all *N*, and for ρ = 0.1 at *N* = 31 and 55. Most important is that if ρ = 0 the expected posterior model probability of snapshot naïve suggests a small effect (ρ = 0.1) up to *N* ≤ 300 (i.e., 600 observations in original study and replication combined). Evidence in favor of a small true effect size is even *increasing* in sample size until *N* = 300, where the expected value of the posterior model probability of incorrect snapshot ρ = 0.1 is .662 and larger than the .338 for the correct snapshot of zero true effect size. Even when *N* = 1,000, evidence in favor of a small effect hardly diminished; the expected posterior model probability (.242) is only little lower than .25. The performance of snapshot naïve is better than snapshot hybrid’s performance for ρ = 0.1 at *N*≥96, and for medium and large true effect size, i.e., expected posterior model probabilities at the correct snapshot are highest for snapshot naïve. Note, however, that for a medium true effect size evidence in favor of a strong effect (ρ_S_ = 0.5) also increases for small sample size (*N* = 31; expected posterior model probability increases from .25 to .417). All these results can be explained by two related consequences of correcting for the statistical significance of the original study.

The first consequence is that not correcting for statistical significance of the original study leads to overestimation of effect size. The last column of Table 2 presents the expected value of fixed-effect meta-analysis, and consequently, its bias. The bias decreases both in true effect size and sample size, and is most severe for ρ = 0 and *N* = 31 (0.215). Bias results in a higher expected value of the posterior model probability of ‘incorrect’ snapshots (ρ_S_≠ρ) for snapshot naïve. The fact that snapshot naïve performs relatively worse for a true small effect than for a medium and strong true effect is thus because overestimation is worse for lower true effect size, particularly for small *N*.

The second consequence is that snapshot hybrid assigns a relatively higher ‘weight’ to the likelihood of the original effect under a zero true effect, compared to snapshot naïve. This is because, in contrast to snapshot naïve, the replication’s likelihood is multiplied by the reciprocal of statistical power (which is the ‘weight’) under snapshot hybrid (see Eq (2) and Fig 1), and statistical power increases in true effect size. In the extreme case, for very large sample size (e.g., *N* > 10,000), the *only* difference between snapshot hybrid and snapshot naïve is that the likelihood of the original effect under a zero hypothesized effect is multiplied by 40 under snapshot hybrid, because the likelihoods at other snapshots are multiplied by 1 under snapshot hybrid (statistical power then equals 1 at these snapshots). The relatively higher weight assigned to the likelihood of the original study’s effect under the hypothesized zero effect explains why snapshot hybrid performs better than snapshot naïve if ρ = 0, for all values of *N*. This relatively higher weight translates into higher posterior model probabilities for ρ_S_ = 0 under snapshot hybrid than snapshot naïve. These higher posterior model probabilities for ρ_S_ = 0 under snapshot hybrid also explain why snapshot naïve outperforms snapshot hybrid for nonzero true effect size in combination with large sample size. For nonzero true effect size and small sample size, however, snapshot hybrid outperforms snapshot naïve because then the adverse effect of overestimation in snapshot naïve is stronger than the higher weight of (incorrect) snapshot ρ_S_ = 0 in snapshot hybrid.

To sum up, sample sizes of 300 for the original study and replication are needed to obtain expected posterior model probabilities with snapshot hybrid close to .75 or higher for a true effect size of ρ = 0, ρ = 0.1, and ρ = 0.3, whereas a sample size of 55 for the original study and replication is sufficient for ρ = 0.5. Hence, small sample sizes (sample size of about 50 per study) are sufficient to make correct decisions if true effect size is large, whereas for zero or small true effect size large sample sizes are required (sample size of at least 300 up to 1,000 per study). Not taking the statistical significance of the original study into account results in worse performance of snapshot naïve when true effect size is zero or small, or when sample sizes are small. Snapshot naïve outperforms snapshot hybrid, i.e. gives higher expected posterior model probabilities for the correct snapshot as well as lower ones for all incorrect snapshots whenever ρ = 0.1 and *N*≥1,000, ρ = 0.3 and *N*≥300, and ρ = 0.5and *N*≥31. However, snapshot naïve is biased as a result of not taking the statistical significance of the original study into account. Its better performance is a consequence of its bias, just as the high statistical power of the fixed-effect meta-analysis for small true effect size is a consequence of its overestimation of effect size. Hence, we advise to use snapshot hybrid rather than snapshot naïve. However, if a researcher is certain that the true effect size is, for instance, large, snapshot naïve may be used since this method outperforms snapshot hybrid in most conditions.

#### Probability of posterior model probability larger than .25 (π>.25) and .75 (π>.75)

Table 3 shows the probability of how often the posterior model probability is larger than .25 (π>.25), i.e. how often the posterior model probability is larger than the prior model probability. The probability of π>.25 of snapshot hybrid at the correct snapshot is at least .776 and approaches one if the sample sizes increases (bold values in the third to sixth columns of Table 3). The same pattern is observed for snapshot naïve, but the probabilities π>.25 at the correct snapshot are smaller for snapshot naïve than snapshot hybrid if ρ = 0, and ρ = 0.1 and *N*<300, and higher for ρ = 0.3 and ρ = 0.5. The lowest probability of π>.25 at the correct snapshot of snapshot naïve is 0.274 for ρ = 0 and *N* = 31. However, both methods’ probabilities of π>.25 at the incorrect snapshot are also substantial and sometimes even larger for the incorrect than for the correct snapshot. For ρ = 0 and *N* = 31, the probability of π>.25 of snapshot hybrid is higher for the incorrect snapshot at ρ_S_ = 0.1 than the correct snapshot (ρ_S_ = 0). The same holds for snapshot naïve at *N* ≤300 if ρ = 0, and at *N*<96 if ρ = 0.1. If the probability of π>.25 is largest for the correct snapshot, the probability of π>.25 at one of the incorrect snapshots can still be substantial. For instance, if ρ = 0 or ρ = 0.1 and *N* = 300, using snapshot hybrid the probability π>.25 is 0.36 for an incorrect snapshot (ρ_S_ = 0.1 or ρ_S_ = 0, respectively). The probability of π>.25 of snapshot naïve is 0.321 for ρ = 0 and *N* = 1,000 at the incorrect snapshot ρ_S_ = 0.1, and 0.495 for ρ = 0.1 and *N* = 96 at ρ_S_ = 0.3. Probabilities of π>.25 at incorrect snapshots also occur for large true effect sizes in combination with small sample sizes. To conclude, a posterior model probability larger than .25 should not be interpreted as evidence in favor of that effect size, but should be interpreted in combination with posterior model probabilities for the other hypothesized effect sizes.

**Table 3:** Probability of posterior model probability larger than .25 of snapshot hybrid and snapshot naïve.

		Snapshot Hybrid				Snapshot Naïve			
	*N*	ρ_S_ = 0	ρ_S_ = 0.1	ρ_S_ = 0.3	ρ_S_ = 0.5	ρ_S_ = 0	ρ_S_ = 0.1	ρ_S_ = 0.3	ρ_S_ = 0.5
ρ = 0	31	**0.855**	0.914	0.22	0.017	**0.274**	0.725	0.757	0.082
	55	**0.897**	0.84	0.107	0.001	**0.364**	0.891	0.473	0.001
	96	**0.926**	0.705	0.03	0	**0.406**	0.97	0.146	0
	300	**0.939**	0.359	0	0	**0.513**	0.882	0	0
	1,000	**0.984**	0.065	0	0	**0.868**	0.321	0	0
ρ = 0.1	31	0.687	**0.87**	0.417	0.063	0.123	**0.512**	0.885	0.206
	55	0.686	**0.891**	0.322	0.007	0.132	**0.677**	0.758	0.015
	96	0.654	**0.895**	0.206	0	0.103	**0.829**	0.495	0
	300	0.36	**0.931**	0.015	0	0.033	**0.992**	0.024	0
	1,000	0.067	**0.982**	0	0	0.007	**0.999**	0	0
ρ = 0.3	31	0.233	0.483	**0.776**	0.385	0.009	0.119	**0.83**	0.656
	55	0.114	0.391	**0.84**	0.252	0.003	0.109	**0.914**	0.338
	96	0.027	0.234	**0.911**	0.104	0	0.081	**0.961**	0.114
	300	0	0.015	**0.995**	0.003	0	0.009	**0.997**	0.003
	1,000	0	0	**1**	0	0	0	**1**	0
ρ = 0.5	31	0.014	0.062	0.471	**0.887**	0	0.004	0.292	**0.97**
	55	0	0.007	0.265	**0.939**	0	0	0.204	**0.963**
	96	0	0	0.105	**0.973**	0	0	0.096	**0.976**
	300	0	0	0.003	**0.999**	0	0	0.003	**0.999**
	1,000	0	0	0	**1**	0	0	0	**1**

ρ denotes the effect size in the population, *N* is the sample size in the original study and replication, and ρ_S_ refers to the snapshots of effect size. Note that the sum of probabilities across four snapshots is sometimes larger than 1, because posterior model probabilities can be larger than .25 for more than snapshot.

Table 4 illustrates the probability of how often the posterior model probability is larger than .75 (π>.75), when evidence can be interpreted as evidence in favor of that true effect. Hence, the probabilities of π>.75 can be interpreted as how often the methods yield the correct conclusion with respect to the magnitude of the true effect size akin to statistical power in null hypothesis significance testing. Table 4 also shows how often inconclusive results (columns named “Inconcl.”) were obtained, indicating that none of the posterior model probabilities were larger than .75. Focusing first on the results of snapshot hybrid (columns three to seven), the probability of making the wrong decision never exceeds 0.065. However, the probability of obtaining inconclusive results is large for *N*≤96 when ρ = 0 (≥ .706) or ρ = 0.1 (≥ .9). The probability of making the correct decision is at least 0.8 (akin to a power of 0.8) for a sample size in between 300 and 1,000 when ρ = 0 or ρ = 0.1, between 96 and 300 when ρ = 0.3, and between 55 and 96 when ρ = 0.5.

**Table 4:** Probability of posterior model probability larger than .75 of snapshot hybrid and snapshot naïve.

		Snapshot Hybrid					Snapshot Naïve				
	*N*	ρ_S_ = 0	ρ_S_ = 0.1	ρ_S_ = 0.3	ρ_S_ = 0.5	Inconcl.	ρ_S_ = 0	ρ_S_ = 0.1	ρ_S_ = 0.3	ρ_S_ = 0.5	Inconcl.
ρ = 0	31	**0.04**	0	0	0	0.96	**0**	0	0	0.002	0.998
	55	**0.142**	0	0.003	0	0.855	**0.001**	0	0.085	0	0.914
	96	**0.291**	0	0.003	0	0.706	**0.008**	0.343	0.021	0	0.628
	300	**0.641**	0.061	0	0	0.298	**0.118**	0.487	0	0	0.395
	1,000	**0.935**	0.016	0	0	0.049	**0.679**	0.132	0	0	0.189
ρ = 0.1	31	0.01	**0**	0	0.002	0.988	0	**0**	0	0.012	0.988
	55	0.033	**0**	0.017	0	0.95	0	**0**	0.263	0.001	0.736
	96	0.057	**0**	0.043	0	0.9	0	**0.288**	0.165	0	0.547
	300	0.065	**0.625**	0.004	0	0.306	0.001	**0.943**	0.007	0	0.049
	1,000	0.018	**0.933**	0	0	0.049	0.001	**0.993**	0	0	0.006
ρ = 0.3	31	0	0	**0**	0.06	0.94	0	0	**0**	0.157	0.843
	55	0	0	**0.115**	0.05	0.835	0	0	**0.513**	0.077	0.41
	96	0	0	**0.645**	0.025	0.33	0	0.006	**0.802**	0.029	0.163
	300	0	0.005	**0.982**	0.001	0.012	0	0.003	**0.988**	0.001	0.008
	1,000	0	0	**1**	0	0	0	0	**1**	0	0
ρ = 0.5	31	0	0	0	**0.498**	0.502	0	0	0	**0.699**	0.301
	55	0	0	0.018	**0.732**	0.25	0	0	0.034	**0.796**	0.17
	96	0	0	0.027	**0.895**	0.078	0	0	0.024	**0.904**	0.072
	300	0	0	0.001	**0.997**	0.002	0	0	0.001	**0.997**	0.002
	1,000	0	0	0	**1**	0	0	0	0	**1**	0

ρ denotes the effect size in the population, *N* is the sample size in the original study and replication, and ρ_S_ refers to snapshots of effect size. The columns “Inconcl.” indicate the probability of observing inconclusive results (i.e., none of the posterior model probabilities for the hypothesized effect sizes was larger than .75)

The probability of making a false decision using snapshot naïve (last five columns) can be substantial for true effect sizes zero to medium. When ρ = 0, the probability of making the false decision that the true effect size is of small magnitude is larger than the probability of drawing the correct conclusion for *N*≤300. The probability of making false decisions are 0.263 for ρ = 0.1 at *N* = 55, and 0.157 for ρ = 0.3 at *N* = 31. The probability of observing inconclusive results with snapshot naïve was large for *N*≤96 when ρ = 0 (≥ .628) or ρ = 0.1 (≥ .547). The probability of making a correct decision does not exceed 0.8 when ρ = 0 for *N*≤1,000, and exceeds 0.8 when ρ = 0.1 and sample size between 96 and 300, and ρ = 0.3 and ρ = 0.5 in combination with sample size between 55 and 96.

To conclude, when true effect size is zero or small, very large sample sizes are required to make correct decisions and snapshot hybrid should be used to take the statistical significance of the original study into account; using snapshot naïve likely results in wrong conclusions when sample size is smaller than 300. When true effect size is medium or large, smaller sample sizes are sufficient to make correct decisions. Snapshot naïve yields both higher probabilities of making correct decisions and lower probabilities of making incorrect decisions when ρ = 0.1 and *N* = 1,000, ρ = 0.3 and *N*≥300, ρ = 0.5 and *N*≥96.

#### Conclusions

The probability of making the correct decision with snapshot hybrid, based on posterior model probabilities larger than .75, is at least 0.8 (akin to a power of 0.8) for a sample size in between 300 and 1,000 when ρ = 0 or ρ = 0.1, between 96 and 300 when ρ = 0.3, and between 55 and 96 when ρ = 0.5. The probability of making a false decision using snapshot naïve can be substantial for true effect sizes zero (even for samples sizes of 300 per group in both studies) to medium. Whereas snapshot hybrid outperforms snapshot naïve if there is no or a small true effect, snapshot naïve generally outperformed snapshot hybrid for medium and large true effect sizes. Importantly, the results of both methods also illustrate that it is hard to obtain conclusive results about the magnitude of the true effect size in situations with sample sizes that are illustrative for current research practice. In the penultimate section of this paper, we use snapshot hybrid to derive the sample size of the replication to obtain evidence of a true effect, akin to power analysis.

## Replicability projects

The Reproducibility Project Psychology (RPP; [5]) and the Experimental Economics Replication Project (EE-RP; [6]) are two projects that studied the replicability of psychological and economic experimental research by replicating published research. Articles for inclusion in RPP were selected from three high impact psychological journals (Journal of Experimental Psychology: Learning, Memory, and Cognition, Journal of Personality and Social Psychology, and Psychological Science) published in 2008. EE-RP included all articles with a between-subject experimental design published in the American Economic Review and the Quarterly Journal of Economics between 2011 and 2014. The most important finding from these articles was selected for both projects to be replicated and the replication was conducted according to a predefined analysis plan in order to ensure that the replication was as close as possible to the original study.

RPP contained 100 studies that were replicated. A requirement for applying snapshot hybrid is that the original study has to be statistically significant. Three observed effect sizes were reported as not being statistically significant in the original studies of RPP. However, of the remaining 97 original effect sizes reported as statistically significant, recalculation of their *p*-values revealed that four were actually not statistically significant either, but slightly larger than .05 [5]; these were excluded as well. The remaining 93 study-pairs included 26 study-pairs that had to be excluded because the correlation coefficient and standard error could not be computed for these study-pairs. This was, for instance, the case for *F*(*df*_1_ >1,*df*_2_) or χ^2^. Hence, the snapshot hybrid and snapshot naïve were applied to 67 study-pairs. EE-RP included 18 study-pairs. The effect size measure of the study-pairs included in EE-RP was also the correlation coefficient. Only two studies had to be excluded because the original study was not statistically significant. Hence, the snapshot hybrid and snapshot naïve were applied to 16 study-pairs of the EE-RP. Information on effect sizes and sample sizes of the study-pairs and the results of the snapshot hybrid and snapshot naïve are reported in S1 Table for EE-RP and S2 Table for RPP.

Table 5 lists the posterior model probabilities averaged over all the study-pairs of the snapshot hybrid and snapshot naïve. The difference between the average posterior model probabilities of snapshot hybrid and snapshot naïve was largest for the study-pairs in RPP at ρ_S_ = 0 (0.293 vs. 0.126). Average posterior model probabilities of both the snapshot hybrid and snapshot naïve based on the study-pairs in EE-RP were larger at ρ_S_ = 0.3 and ρ_S_ = 0.5 than the study-pairs in RPP, whereas this was the other way around at ρ_S_ = 0 and ρ_S_ = 0.1.

**Table 5:** Average posterior model probabilities for the study-pairs in EE-RP and RPP for snapshot hybrid and snapshot naïve at four different snapshots (ρ_S_ = 0; 0.1; 0.3; 0.5).

		ρ_S_			
		0	0.1	0.3	0.5
Snapshot Hybrid	EE-RP	0.084	0.137	0.34	0.44
	RPP	0.293	0.234	0.217	0.256
Snapshot Naïve	EE-RP	0.03	0.165	0.361	0.444
	RPP	0.126	0.285	0.267	0.321

Table 6 shows the proportions of how often the posterior model probability was larger than .25 for the snapshot hybrid and snapshot naïve. Seven different categories were used because the posterior model probability could be larger than .25 for two snapshots. A study-pair was assigned to one of the categories belonging to snapshots ρ_S_ = 0, 0.1, 0.3, and 0.5 if the posterior model probability of only one of these snapshots was larger than .25. If the posterior model probability of a study-pair was, for instance, 0.4 of snapshot ρ_S_ = 0 and 0.5 of snapshot ρ_S_ = 0.1, the study-pair was assigned to category 0–0.1. The proportion of study-pairs in the categories ρ_S_ = 0 and 0–0.1 was larger for snapshot hybrid than snapshot naïve. On the contrary, snapshot naïve resulted in a higher proportion of study-pairs in the categories ρ_S_ = 0.3, 0.3–0.5 and 0.5 than snapshot hybrid. The large proportions for the categories including two snapshots (e.g., 0–0.1) indicated that drawing definite conclusions about the magnitude of the effect size was often impossible. Comparing the results between EE-RP and RPP shows that the proportion of study-pairs in the categories ρ_S_ = 0 and 0–0.1 was larger for RPP than EE-RP. The proportion of study-pairs in the categories ρ_S_ = 0.3, 0.3–0.5, and 0.5 was larger for EE-RP than RPP.

**Table 6:** Proportions of how often the posterior model probability is larger than .25 for the study-pairs in EE-RP and RPP for snapshot hybrid and snapshot naïve at seven different snapshots (ρ_S_ = 0; 0–0.1; 0.1; 0.1–0.3; 0.3; 0.3–0.5; 0.5).

		ρ_S_						
		0	0–0.1	0.1	0.1–0.3	0.3	0.3–0.5	0.5
Snapshot Hybrid	EE-RP	0	0.125	0.062	0.062	0.312	0	0.438
	RPP	0.134	0.284	0.045	0.119	0.06	0.164	0.194
Snapshot Naïve	EE-RP	0	0.062	0.125	0	0.375	0	0.438
	RPP	0.015	0.194	0.149	0.06	0.164	0.179	0.239

Table 7 presents the proportions of how often the posterior model probability was larger than .75 for the snapshot hybrid and snapshot naïve for snapshots ρ_S_ = 0, 0.1, 0.3, and 0.5. None of the posterior model probabilities at the different snapshots was larger than .75 for snapshot hybrid in 18.8% and 62.7% of the study-pairs in EE-RP and RPP (last column of Table 7, respectively. For snapshot naïve, no posterior model probability was larger than .75 in 6.3% of the study-pairs in EE-RP and 52.2% of the study-pairs in RPP. Hence for most of the effects studied in the RPP, no decisions can be made on the magnitude of the true effect size, whereas for EE-RP decisions can be made in the majority of effects studied.

**Table 7:** Proportions of how often the posterior model probability is larger than .75 for the study-pairs in EE-RP and RPP for snapshot hybrid and snapshot naïve at four different snapshots (ρ_S_ = 0; 0.1; 0.3; 0.5) and how often none of the posterior model probabilities is larger than .75 (Inconclusive results, final column).

		ρ_S_				
		0	0.1	0.3	0.5	Inconcl.
Snapshot Hybrid	EE-RP	0	0.062	0.312	0.438	0.188
	RPP	0.134	0.030	0.045	0.164	0.627
Snapshot Naïve	EE-RP	0	0.125	0.375	0.438	0.062
	RPP	0.015	0.119	0.104	0.239	0.522

For the EE-RP, no evidence in favor of a zero true effect was obtained, whereas the majority of effects examined showed evidence in favor of a medium (31.2% for snapshot hybrid and 37.5% for snapshot naïve) or large true effect (43.8% for both snapshot hybrid and snapshot naïve). In RPP, evidence in favor of the null hypothesis was obtained for 13.4% of the effects examined according to snapshot hybrid. This number is much lower than the percentage of statistically nonsignificant replications in RPP (73.1%). A small percentage of study-pairs obtained evidence in favor of a large true effect (16.4%-23.9%).

The studies in RPP can be divided into social and cognitive psychology studies. The proportions of how often the posterior model probability was larger than .75 for social psychology and cognitive psychology is presented in Table 8. According to snapshot hybrid, the true effect size was more often zero in studies in social psychology than in cognitive psychology (23.5% vs. 3.0%), whereas it was more often large in cognitive psychology than in social psychology (21.2% vs 11.8%). For approximately half of the study-pairs in both fields, none of the posterior model probabilities of snapshot hybrid and snapshot naïve was larger than .75 (final column of Table 8).

**Table 8:** Proportions of how often the posterior model probability is larger than .75 for the study-pairs in RPP grouped by social and cognitive psychological studies for snapshot hybrid and snapshot naïve at four different snapshots (ρ_S_ = 0; 0.1; 0.3; 0.5) and how often none of the posterior model probabilities is larger than .75 (Inconclusive results, final column).

		ρ_S_				
		0	0.1	0.3	0.5	Inconcl.
Snapshot Hybrid	Social	0.235	0.059	0	0.118	0.588
	Cognitive	0.030	0	0.091	0.212	0.667
Snapshot Naïve	Social	0.029	0.176	0.059	0.118	0.618
	Cognitive	0	0.061	0.152	0.364	0.424

## Determining sample size of replication with snapshot hybrid

Snapshot hybrid can also be used for computing the required sample size where *P*(π_x_≥*a*) = *b* with *a* being the desired posterior model probability and *b* the desired probability for a correct decision (i.e., desired probability of observing a posterior model probability larger than *a*). Computing the required sample size with snapshot hybrid is akin to computing the required sample size with a power analysis in null hypothesis significance testing. A value for *a* is 0.75 that corresponds to a Bayes Factor of 3 [26] and *b* equal to 0.8 reflecting 80% statistical power. Note that any other desired values for *a* and *b* can be chosen. We do not compute the required sample size with snapshot naïve because it falsely does not take the significance of the original study into account and is unsuitable for ρ = 0.

For computing the required sample size of the replication, we need information on the effect size or test statistic and sample size(s) of the original study and the expected true effect size in the population. The four different hypothesized effect sizes or snapshots (zero, small, medium, large) are used as before. *P*(π_x_≥*a*) for the hypothesized effect size is calculated using numerical integration. The required sample size of the replication can be obtained by optimizing the sample size until *b* is obtained. The required sample size of the replication is also computed when the original study is ignored. A researcher may opt to ignore information of the original study if he or she believes that the original study does not estimate the same true effect or has other reasons to discard this information.

The procedure for determining the sample size of the replication is programmed in R and requires as input the observed effect size and sample size of the original study, α-level, desired posterior model probability (*a*), and desired probability (*b*). Users can also specify (besides specifying the α-level, *a*, and *b*) the two group means, standard deviations, and sample sizes or a *t*-value and sample sizes in order to compute the required sample size in case of a two-independent groups design. The output is a 4×2 table with for each hypothesized effect size the required total replication sample size when the original effect size is included or excluded. An easy to use web application is available to compute the required sample size for researchers who are not familiar with R (https://rvanaert.shinyapps.io/snapshot/).

Determining the sample size of the replication with snapshot hybrid for the example by Maxwell et al. [8] with *r*_*o*_ = .243 and *N* = 80 resulted in the sample sizes presented in Table 9 for each hypothesized effect size, using *a* = 0.75 and *b* = 0.8. Higher sample sizes are needed for zero and small hypothesized effect size than for medium and strong hypothesized effect size. When ignoring the original study, *less* observations are needed for nonzero hypothesized effect sizes than after incorporating the original study. The reason is that, after taking the statistical significance of the original effect into account, the original effect provides evidence in favor of a zero true effect. This is also the reason that *more* observations are needed for a zero hypothesized effect size when the original study is ignored (*N* = 645) relative to incorporating it (*N* = 587). We note that Maxwell et al. [8] conducted a power analysis based on the results of the original study to compute the required sample size in the replication and ended up with a sample size of 172. The explanation for their low required sample size is that they likely overestimate effect size with the original study by not taking its statistical significance into account. Sample size of the replication obtained with snapshot hybrid may also be larger than the sample size obtained with a power analysis, because four different hypothesized effect sizes are examined with snapshot hybrid instead of one in a usual power analysis. However, this will only have a minor influence since the posterior model probability for at least two out of four effect sizes will be small if the sample size is large.

**Table 9:** Required sample size computed with snapshot hybrid based on characteristics of the original study as described in Maxwell et al. [8]; *r*_*o*_ = .243 and *N* = 80.

	With original study	Without original study
ρ_S_ = 0	587	645
ρ_S_ = 0.1	709	664
ρ_S_ = 0.3	223	215
ρ_S_ = 0.5	284	116

Sample size was computed with snapshot hybrid for a desired posterior model probability of *a* = 0.75 and the desired probability of observing a posterior model probability larger than *a* was *b* = 0.8. The hypothesized effect size was equal to ρ_S_ = 0 (no effect), 0.1 (small), 0.3 (medium), and 0.5 (large). The penultimate column refers to the required sample size where information of the original study is included and the last column where this information is excluded.

Finally, we emphasize that snapshot hybrid is sensitive to the observed effect size in the original study. An observed effect size in the original study close to a hypothesized effect size results in a smaller required sample size for the replication than if the observed effect size substantially deviates from the hypothesized effect size. Our web application can be used to examine the sensitivity of the required sample size to the results of the original study. This provides information on how much evidence there is for a particular hypothesized effect size in the original study after taking into account statistical significance in this study. The first column is affected by the statistics of the original study, whereas the last is not because it ignores the original study. For instance, if Maxwell et al. [8] had postulated *r*_*o*_ = .243 and *N* = 800, the required sample size for the replication by taking into account the information of the original study is 3,691, 561, a sample size of less than 4, and 1,521 for ρ = 0; 0.1; 0.3; 0.5, respectively. The reason that only very few observations are required for medium hypothesized effect size and very large sample size for zero and large hypothesized effect size is that the original study provides strong evidence of a close to medium true effect size.

## Conclusion and discussion

The high number of statistical significant findings in the literature (e.g., [1–3]) does not match the average low statistical power [28–30], and raises concerns about the reliability of published findings. Several projects recently systematically replicated published studies in medicine [4], psychology (RPP; [5]), and economics (EE-RP; [6]) to examine their replicability. Characteristic of all these projects is that most effects were originally statistically significant, but not significant in the replication. Problems with traditional methods to analyze these results are that (1) NHST is not informative for the magnitude of the true effect size, (2) no evidence can be obtained for a true zero effect, and (3) they do not take into account the statistical significance of the original study. To solve these problems we developed a method (snapshot Bayesian hybrid meta-analysis method, snapshot hybrid for short) that computes the posterior model probability for a set of effect sizes (no, small, medium, and large effect) by statistically combining the original study and replication, while at the same time taking the statistical significance of the original study into account. Desirable properties of snapshot hybrid are its few assumptions, its straightforward interpretation as the probability that the true effect size is zero, small, medium or large, and the ease with which posterior model probabilities can be recalculated using different sets of prior model probabilities.

Researchers can apply snapshot hybrid with the R function “snapshot” in the “puniform” package (package can be installed with the following R code devtools::install_github(“RobbievanAert/puniform”). The “req.ni.r” function which is also in the “puniform” package can be used for computing the required sample size of the replication to achieve a certain posterior model probability for hypothesized effect sizes equal to zero, small, medium, and large, akin to power analysis. Researchers not familiar with R can use the web application (https://rvanaert.shinyapps.io/snapshot/) for applying snapshot hybrid and computing the required sample size of the replication.

We examined the performances of snapshot hybrid and a method that does not take into account that the original study is statistically significant (snapshot naïve). Our analysis shows that snapshot naïve hardly ever can provide evidence in favor of a true zero effect; even if both original and replication effect have a sample size of 1,000, the expected posterior model probability in favor of a small effect is close to the prior model probability of .25. Hence, we recommend not using any method that does not take into account statistical significance of the original study (including fixed-effect meta-analysis) when the goal is examining if a nonzero true effect size exists. Snapshot naïve outperformed snapshot hybrid for medium true effect size and sample sizes of 300 per study, and for large true effect size and sample sizes of 31. Thus, we only recommend using methods that do not correct for statistical significance in the original study when true effect size is strongly suspected to be large, or medium in combination with large sample sizes (> 300) of both the original study and the replication.

By taking the statistical significance of the original effect into account snapshot hybrid yields accurate evaluations of not only zero true effect size, but larger true effect size as well. The probability of making the correct decision with snapshot hybrid, based on posterior probabilities larger than .75, is at least 0.8 (akin to a power of 0.8) for sample sizes in between 300 and 1,000 when ρ = 0 or ρ = 0.1, between 96 and 300 when ρ = 0.3, and between 55 and 96 when ρ = 0.5. Due to its accurate evaluations, particularly if true effect size is zero, we recommend using snapshot hybrid when evaluating effect size based on a statistically significant original study and a replication. Importantly, our results also confirm previous research (e.g., [8, 22]) that it is hard to obtain conclusive results about the magnitude of the true effect size in situations with sample sizes that are illustrative for current research practice.

Several conclusions can be drawn from the application of snapshot hybrid to the data of RPP and EE-RP. First, in the majority of study-pairs in RPP no evidence was found for any of the true effects, as opposed to in EE-RP where evidence was found for one true effect size considering a zero, small, medium, or large true effect size in about 80% of the study-pairs. This shows that sample sizes of the original study and replication in RPP were generally often not large enough to draw definite conclusions about the magnitude of the true effect size (e.g., [8, 22]). Second, true effect size was generally higher in EE-RP than in RPP. However, evidence in favor of the null hypothesis was found for only 13.4% of the study-pairs in RPP, as opposed to the much higher percentage of statistically nonsignificant replications in RPP (73.1%). This is in line with the argumentation of Maxwell et al. [8], who argue that sample sizes of the replications in RPP are generally too small to draw conclusions on the absence of a true effect. Finally, within RPP true effect size was generally lower for study-pairs in social than cognitive psychology.

Our study and snapshot hybrid have several limitations. First, we analytically evaluated the statistical properties of the snapshot hybrid and snapshot naïve by assuming equal sample sizes of the original study and replication. Most often their sample sizes are somewhat different, with the replication generally having larger sample size than the original study. Hence, our results on statistical properties should be considered as illustrations of the effect of sample size on the performance of both snapshot naïve and snapshot hybrid. Note that our web application can be used to examine what the effect is of different sample sizes of the original study when calculating the required replication sample size.

A limitation of snapshot hybrid seems to be the requirement that the original study is statistically significant. However, most studies in the social sciences contain statistically significant results; about 95% of the studies in psychology contain significant results (e.g., [1, 3]) and 97% and 89% of the original findings in RPP [5] and EE-RP [6] were statistically significant. Another apparent limitation of snapshot hybrid is that it assumes that the same true effect is underlying the original study and replication. However, an exact replication is highly similar to an original study and no or a small amount of heterogeneity in true effect size may be expected. Furthermore, two studies are not sufficient to estimate the amount of heterogeneity (e.g., [12, 31]).

Our current implementation of snapshot hybrid assumes discrete values of hypothesized effect size, rather than distributions of hypothesized effect size as in continuous Bayesian analyses. A disadvantage of using discrete values is that if the true effect size is in between these values, the results of our analysis on statistical properties do no longer apply. That is, higher samples sizes are needed to obtain evidence in favor of the discrete value closest to the actual true effect size. Other hypothesized effect sizes can be used as a sort of sensitivity analysis to examine whether the true effect size is in between the originally proposed hypothesized effect sizes. For example, if the true effect size is ρ = 0.2 and thus between ρ = 0.1 and ρ = 0.3, the highest posterior model probability will be observed for ρ = 0.2 when hypothesized effect sizes ρ = 0, ρ = 0.1, ρ = 0.2, and ρ = 0.3 are chosen. Snapshot hybrid could also be implemented using intervals of hypothesized effect size, say 0, 0–0.1, 0.1–0.3, 0.3–0.5, > 0.5, while keeping all of its desirable properties except for one: The posterior model probabilities can no longer be easily updated using Eq (4) when assuming other than equal prior model probabilities. However, we chose for discrete hypothesized effect size values in the current implementation of snapshot hybrid because we believe most researchers think in terms of zero, small, medium, and large effect size, and wish to carry out power analyses assuming these effect sizes as in our web application.

Another limitation of snapshot hybrid in its current implementation is that it can only deal with one (statistically significant) original study and one replication. Including more than one original study or replication will usually yield more divergence in the posterior model probabilities of the set of effect sizes and enable researchers to draw more reliable conclusions. We will extend the current snapshot hybrid method such that it can deal with multiple original studies and replications in the future. A final limitation is that the results of snapshot hybrid will be biased in case of questionable research practices or *p*-hacking in the original study. Questionable research practices bias the *p*-values (e.g., [15, 18, 19, 32, 33]) and therefore also the truncated density of the original study. The extent to which the results of snapshot hybrid becomes biased due to questionable research practices may be subject for further study. We note, however, that *no* existing method can deal with questionable research practices.

To conclude, the unrealistic high rate of statistically significant findings in the published literature and the results of RPP and EE-RP suggest that the literature is distorted with false positive findings and too high effect size estimates. We propose and recommend snapshot hybrid for evaluating the magnitude of the true effect size underlying an original study and replication that computes the posterior model probability for a zero, small, medium, and large hypothesized effect. The method has the advantage over other existing methods, because it is the first method that adjusts for publication bias by taking statistical significance of the original study into account. Moreover, the method can also be used for determining the sample size in the replication akin to power analysis in NHST. The snapshot hybrid method is easy to understand and to apply and provides useful insights in evaluating an original study and replication.

## Supporting information

S1 Table Correlation coefficients (*r*), sample sizes (*n*), and *p*-values (*p*) of the original study and replication of the study-pairs in EE-RP and results of the snapshot hybrid and snapshot naïve at four different snapshots (ρ_S_ = 0; 0.1; 0.3; 0.5). (DOCX)

S2 Table Correlation coefficients (*r*), sample sizes (*n*), and *p*-values (*p*) of the original study and replication of the study-pairs in RRP and results of the snapshot hybrid and snapshot naïve at four different snapshots (ρ_S_ = 0; 0.1; 0.3; 0.5). (DOCX)

## References

[1] Fanelli D. Negative results are disappearing from most disciplines and countries. Scientometrics. 2012;90(3):891–904.

[2] Fanelli D. Do pressures to publish increase scientists' bias? An empirical support from US States Data. PLoS ONE. 2010;5(4). 10.1371/journal.pone.0010271 PMC2858206 20422014

[3] Sterling TD, Rosenbaum WL, Weinkam JJ. Publication decisions revisited: The effect of the outcome of statistical tests on the decision to publish and vice versa. The American Statistician. 1995;49(1):108–12.

[4] Begley CG, Ellis LM. Drug development: Raise standards for preclinical cancer research. Nature. 2012;483(7391):531–3. 10.1038/483531a 22460880

[5] Open Science Collaboration. Estimating the reproducibility of psychological science. Science. 2015;349(6251). 10.1126/science.aac4716 26315443

[6] Camerer CF, Dreber A, Forsell E, Ho TH, Huber J, Johannesson M, et al Evaluating replicability of laboratory experiments in economics. Science. 2016;351(6280):1433–6. 10.1126/science.aaf0918 26940865

[7] Gilbert DT, King G, Pettigrew S, Wilson TD. Comment on "Estimating the reproducibility of psychological science". 2016;351(6277). 10.1126/science.aad7243 26941311

[8] Maxwell SE, Lau MY, Howard GS. Is psychology suffering from a replication crisis? What does "failure to replicate" really mean? American Psychologist. 2015;70(6):487–98. 10.1037/a0039400. 26348332

[9] Anderson SF, Maxwell SE. There's more than one way to conduct a replication study: Beyond statistical significance. Psychological Methods. 2016;21(1):1–12. 10.1037/met0000051 26214497

[10] Borenstein M, Hedges LV, Higgins JPT, Rothstein HR. Introduction to meta-analysis. Chichester, UK: John Wiley & Sons, Ltd.; 2009.

[11] Wagenmakers E-J. A practical solution to the pervasive problems of p values. Psychonomic Bulletin & Review. 2007;14(5):779–804. 18087943 10.3758/bf03194105

[12] Borenstein M, Hedges LV, Higgins JPT, Rothstein HR. A basic introduction to fixed-effect and random-effects models for meta-analysis. Research Synthesis Methods. 2010;1(2):97–111. 10.1002/jrsm.12 26061376

[13] Ioannidis JP. Why most discovered true associations are inflated. Epidemiology. 2008;19(5):640–8. 10.1097/EDE.0b013e31818131e7 18633328

[14] Lane DM, Dunlap WP. Estimating effect size: Bias resulting from the significance criterion in editorial decisions. British Journal of Mathematical & Statistical Psychology. 1978;31:107–12.

[15] van Assen MALM, van Aert RCM, Wicherts JM. Meta-analysis using effect size distributions of only statistically significant studies. Psychological Methods. 2015;20(3):293–309. 10.1037/met0000025. 25401773

[16] Rothstein HR, Sutton AJ, Borenstein M. Publication bias in meta-analysis In: Rothstein HR, Sutton AJ, Borenstein M, editors. Publication bias in meta-analysis: Prevention, assessment and adjustments. Chichester, UK: Wiley; 2005.

[17] Neuliep JW, Crandall R. Everyone was wrong—There are lots of replications out there. Journal of Social Behavior and Personality. 1993;8(6):1–8.

[18] van Aert RCM, Wicherts JM, van Assen MALM. Conducting meta-analyses on p-values: Reservations and recommendations for applying p-uniform and p-curve. Perspectives on Psychological Science. 2016;11(5):713–29. 10.1177/1745691616650874 27694466 PMC5117126

[19] Simonsohn U, Nelson LD, Simmons JP. P-curve and effect size: Correcting for publication bias using only significant results. Perspectives on Psychological Science. 2014;9(6):666–81. 10.1177/1745691614553988 26186117

[20] McShane BB, Böckenholt U, Hansen KT. Adjusting for publication bias in meta-analysis: An evaluation of selection methods and some cautionary notes. Perspectives on Psychological Science. 2016;11(5):730–49. 10.1177/1745691616662243 27694467

[21] van Aert RCM, van Assen MALM. Examining reproducibility in psychology: A hybrid method for statistically combining a biased original study and replication. Manuscript submitted for publication2016. 10.3758/s13428-017-0967-6 PMC6096648 28936638

[22] Etz A, Vandekerckhove J. A Bayesian perspective on the Reproducibility Project: Psychology. PLoS ONE. 2016;11(2). 10.1371/journal.pone.0149794 PMC4769355 26919473

[23] Fisher RA. On the 'probable error' of a coefficient of correlation deduced from a small sample. Metron. 1921;1:3–32.

[24] Cohen J. Statistical power analysis for the behavioral sciences. 2nd ed Hillsdale NJ: Lawrence Erlbaum Associates; 1988.

[25] Raudenbush SW. Analyzing effect sizes: Random-effects models In: Cooper H, Hedges LV, Valentine JC, editors. The Handbook of Research Synthesis and Meta-Analysis. New York: Russell Sage Foundation; 2009 p. 295–315.

[26] Kass RE, Raftery AE. Bayes factors. Journal of the American Statistical Association. 1995;90(430):791.

[27] R Core Team. R: A language and environment for statistical computing. Vienna, Austria: R Foundation for Statistical Computing; 2017.

[28] Bakker M, van Dijk A, Wicherts JM. The rules of the game called psychological science. Perspectives on Psychological Science. 2012;7(6):543–54. 10.1177/1745691612459060 26168111

[29] Cohen J. Things I have learned (so far). American Psychologist. 1990;45(12):1304–12.

[30] Button KS, Ioannidis JP, Mokrysz C, Nosek BA, Flint J, Robinson ES, et al Power failure: Why small sample size undermines the reliability of neuroscience. Nature Reviews Neuroscience. 2013;14(5):365–76. 10.1038/nrn3475 23571845

[31] IntHout J, Ioannidis JP, Borm GF. The Hartung-Knapp-Sidik-Jonkman method for random effects meta-analysis is straightforward and considerably outperforms the standard DerSimonian-Laird method. BMC Medical Research Methodology. 2014;14. 10.1186/1471-2288-14-25 PMC4015721 24548571

[32] Bruns SB, Ioannidis JP. P-curve and p-hacking in observational research. PLoS ONE. 2016;11(2)::e0149144 10.1371/journal.pone.0149144 26886098 PMC4757561

[33] Ulrich R, Miller J. p-hacking by post hoc selection with multiple opportunities: Detectability by skewness test?: Comment on Simonsohn, Nelson, and Simmons (2014). Journal of Experimental Psychology: General. 2015;144(6):1137–45. 26595841 10.1037/xge0000086

