# Supplementary material for: Bayesian evaluation of effect size after replicating an original study
Source: PLoS One. 2017 Apr 7;12(4):e0175302. doi: 10.1371/journal.pone.0175302 (PMC5384677; doi:10.1371/journal.pone.0175302)
Supplement: S1 Table — (DOCX) [file pone.0175302.s001.docx]

|  |  |  |  |  |  |  | **Snapshot Hybrid** | | | | **Snapshot Naïve** | | | |
| --- | --- | --- | --- | --- | --- | --- | --- | --- | --- | --- | --- | --- | --- | --- |
| ID | *r_o_* | *n_o_* | *p_o_* | *r_r_* | *n_r_* | *p_r_* | ρ_S_=0 | ρ_S_=0.1 | ρ_S_=0.3 | ρ_S_=0.5 | ρ_S_=0 | ρ_S_=0.1 | ρ_S_=0.3 | ρ_S_=0.5 |
| 33 | 0.18 | 120 | 0.049 | 0.08 | 318 | 0.155 | 0.386 | 0.614 | 0 | 0 | 0.076 | 0.924 | 0 | 0 |
| 34 | 0.31 | 117 | 0.001 | 0.23 | 357 | 0 | 0 | 0.034 | 0.966 | 0 | 0 | 0.007 | 0.993 | 0 |
| 35 | 0.72 | 216 | 0 | 0.66 | 360 | 0 | 0 | 0 | 0 | 1 | 0 | 0 | 0 | 1 |
| 36 | 0.38 | 162 | 0 | 0.36 | 264 | 0 | 0 | 0 | 0.988 | 0.012 | 0 | 0 | 0.988 | 0.012 |
| 37 | 0.84 | 72 | 0 | 0.17 | 168 | 0.027 | 0 | 0 | 0.07 | 0.93 | 0 | 0 | 0.052 | 0.948 |
| 39 | 0.76 | 54 | 0 | -0.12 | 96 | 1.755 | 0.063 | 0.379 | 0.557 | 0.002 | 0.004 | 0.107 | 0.884 | 0.004 |
| 40 | 0.72 | 168 | 0 | 0.73 | 128 | 0 | 0 | 0 | 0 | 1 | 0 | 0 | 0 | 1 |
| 41 | 0.21 | 112 | 0.026 | 0.12 | 262 | 0.052 | 0.163 | 0.835 | 0.002 | 0 | 0.026 | 0.962 | 0.012 | 0 |
| 42 | 0.45 | 60 | 0 | 0.31 | 102 | 0.001 | 0 | 0.016 | 0.89 | 0.094 | 0 | 0.003 | 0.859 | 0.138 |
| 43 | 0.64 | 78 | 0 | 0.44 | 40 | 0.004 | 0 | 0 | 0.002 | 0.998 | 0 | 0 | 0.002 | 0.998 |
| 44 | 0.3 | 124 | 0.001 | 0.33 | 128 | 0 | 0 | 0.009 | 0.989 | 0.002 | 0 | 0.002 | 0.996 | 0.002 |
| 45 | 0.83 | 120 | 0 | 0.37 | 160 | 0 | 0 | 0 | 0 | 1 | 0 | 0 | 0 | 1 |
| 46 | 0.28 | 58 | 0.033 | -0.01 | 131 | 1.09 | 0.723 | 0.276 | 0 | 0 | 0.367 | 0.628 | 0.005 | 0 |
| 47 | 0.49 | 288 | 0 | 0.34 | 48 | 0.018 | 0 | 0 | 0.002 | 0.998 | 0 | 0 | 0.002 | 0.998 |
| 48 | 0.66 | 120 | 0 | 0.53 | 220 | 0 | 0 | 0 | 0 | 1 | 0 | 0 | 0 | 1 |
| 49 | 0.32 | 126 | 0 | 0.3 | 90 | 0.004 | 0.001 | 0.026 | 0.969 | 0.004 | 0 | 0.006 | 0.99 | 0.005 |
| Mean | | | | | | | 0.084 | 0.137 | 0.34 | 0.44 | 0.03 | 0.165 | 0.361 | 0.444 |
