# Supplementary material for: Bayesian evaluation of effect size after replicating an original study
Source: PLoS One. 2017 Apr 7;12(4):e0175302. doi: 10.1371/journal.pone.0175302 (PMC5384677; doi:10.1371/journal.pone.0175302)
Supplement: S2 Table — (DOCX) [file pone.0175302.s002.docx]

|  |  |  |  |  |  |  | **Snapshot Hybrid** | | | | **Snapshot Naïve** | | | |
| --- | --- | --- | --- | --- | --- | --- | --- | --- | --- | --- | --- | --- | --- | --- |
| ID | *r_o_* | *n_o_* | *p_o_* | *r_r_* | *n_r_* | *p_r_* | ρ_S_=0 | ρ_S_=0.1 | ρ_S_=0.3 | ρ_S_=0.5 | ρ_S_=0 | ρ_S_=0.1 | ρ_S_=0.3 | ρ_S_=0.5 |
| 1 | 0.595 | 15 | 0.018 | 0.148 | 30 | 0.437 | 0.298 | 0.392 | 0.273 | 0.036 | 0.077 | 0.216 | 0.527 | 0.179 |
| 2 | 0.611 | 25 | 0.001 | 0.23 | 25 | 0.273 | 0.074 | 0.174 | 0.454 | 0.298 | 0.005 | 0.032 | 0.375 | 0.588 |
| 3 | 0.425 | 26 | 0.03 | -0.215 | 33 | 0.231 | 0.701 | 0.284 | 0.014 | 0 | 0.418 | 0.472 | 0.109 | 0.001 |
| 4 | 0.229 | 192 | 0.001 | -0.006 | 270 | 0.92 | 0.613 | 0.387 | 0 | 0 | 0.124 | 0.876 | 0 | 0 |
| 5 | 0.461 | 33 | 0.006 | 0.135 | 49 | 0.358 | 0.249 | 0.45 | 0.293 | 0.009 | 0.038 | 0.216 | 0.701 | 0.045 |
| 6 | 0.595 | 25 | 0.001 | 0.396 | 33 | 0.022 | 0.011 | 0.048 | 0.377 | 0.563 | 0.001 | 0.006 | 0.217 | 0.776 |
| 7 | 0.715 | 101 | 0 | 0.131 | 16 | 0.317 | 0 | 0 | 0 | 1 | 0 | 0 | 0 | 1 |
| 8 | 0.561 | 39 | 0 | -0.111 | 33 | 0.543 | 0.287 | 0.418 | 0.279 | 0.017 | 0.038 | 0.195 | 0.684 | 0.083 |
| 10 | 0.699 | 30 | 0 | 0.781 | 31 | 0 | 0 | 0 | 0.002 | 0.998 | 0 | 0 | 0.001 | 0.999 |
| 11 | 0.672 | 23 | 0 | 0.466 | 31 | 0.007 | 0.002 | 0.011 | 0.186 | 0.802 | 0 | 0.001 | 0.086 | 0.912 |
| 15 | 0.195 | 96 | 0.028 | 0.247 | 243 | 0 | 0.003 | 0.282 | 0.715 | 0 | 0 | 0.097 | 0.902 | 0 |
| 19 | 0.56 | 33 | 0.001 | 0.402 | 21 | 0.071 | 0.013 | 0.046 | 0.315 | 0.625 | 0 | 0.006 | 0.188 | 0.806 |
| 20 | 0.224 | 96 | 0.028 | 0.019 | 108 | 0.842 | 0.627 | 0.371 | 0.002 | 0 | 0.204 | 0.775 | 0.021 | 0 |
| 24 | 0.364 | 154 | 0 | 0.284 | 50 | 0.045 | 0 | 0.006 | 0.959 | 0.035 | 0 | 0.002 | 0.963 | 0.036 |
| 27 | 0.378 | 33 | 0.029 | 0.38 | 72 | 0.001 | 0.007 | 0.068 | 0.755 | 0.17 | 0 | 0.012 | 0.665 | 0.323 |
| 29 | 0.738 | 9 | 0.021 | 0.704 | 16 | 0.002 | 0.012 | 0.035 | 0.208 | 0.745 | 0.001 | 0.007 | 0.105 | 0.887 |
| 32 | 0.623 | 38 | 0 | 0.481 | 39 | 0.002 | 0 | 0.001 | 0.066 | 0.933 | 0 | 0 | 0.034 | 0.966 |
| 33 | 0.517 | 41 | 0 | 0.316 | 41 | 0.044 | 0.01 | 0.058 | 0.533 | 0.399 | 0 | 0.008 | 0.406 | 0.586 |
| 36 | 0.714 | 22 | 0 | 0.682 | 22 | 0 | 0 | 0.001 | 0.045 | 0.954 | 0 | 0 | 0.019 | 0.981 |
| 37 | 0.551 | 13 | 0.05 | 0.35 | 19 | 0.144 | 0.193 | 0.282 | 0.353 | 0.173 | 0.033 | 0.096 | 0.39 | 0.482 |
| 44 | 0.352 | 69 | 0.003 | 0.15 | 178 | 0.045 | 0.078 | 0.745 | 0.177 | 0 | 0.009 | 0.424 | 0.567 | 0 |
| 48 | 0.225 | 94 | 0.029 | -0.052 | 194 | 0.469 | 0.898 | 0.102 | 0 | 0 | 0.581 | 0.419 | 0 | 0 |
| 49 | 0.378 | 36 | 0.022 | -0.03 | 88 | 0.779 | 0.674 | 0.322 | 0.004 | 0 | 0.373 | 0.593 | 0.034 | 0 |
| 52 | 0.206 | 133 | 0.017 | 0.094 | 113 | 0.323 | 0.387 | 0.601 | 0.012 | 0 | 0.067 | 0.859 | 0.075 | 0 |
| 53 | 0.377 | 33 | 0.03 | 0.077 | 75 | 0.514 | 0.456 | 0.496 | 0.048 | 0 | 0.164 | 0.563 | 0.273 | 0 |
| 56 | 0.379 | 101 | 0 | -0.042 | 40 | 0.798 | 0.107 | 0.352 | 0.537 | 0.003 | 0.005 | 0.111 | 0.877 | 0.007 |
| 58 | 0.167 | 184 | 0.023 | 0.037 | 280 | 0.541 | 0.646 | 0.354 | 0 | 0 | 0.144 | 0.856 | 0 | 0 |
| 61 | 0.22 | 110 | 0.021 | -0.005 | 222 | 0.944 | 0.788 | 0.212 | 0 | 0 | 0.342 | 0.658 | 0 | 0 |
| 63 | 0.274 | 70 | 0.021 | 0.074 | 147 | 0.375 | 0.431 | 0.563 | 0.006 | 0 | 0.124 | 0.829 | 0.047 | 0 |
| 65 | 0.432 | 43 | 0.003 | 0.012 | 133 | 0.894 | 0.537 | 0.459 | 0.004 | 0 | 0.232 | 0.735 | 0.033 | 0 |
| 68 | 0.186 | 118 | 0.044 | 0.003 | 224 | 0.964 | 0.814 | 0.186 | 0 | 0 | 0.367 | 0.633 | 0 | 0 |
| 71 | 0.222 | 375 | 0 | 0.073 | 177 | 0.332 | 0.018 | 0.962 | 0.02 | 0 | 0.001 | 0.958 | 0.041 | 0 |
| 72 | 0.208 | 259 | 0.001 | 0.044 | 249 | 0.485 | 0.212 | 0.788 | 0 | 0 | 0.018 | 0.981 | 0 | 0 |
| 81 | 0.268 | 92 | 0.009 | -0.102 | 139 | 0.234 | 0.869 | 0.131 | 0 | 0 | 0.517 | 0.483 | 0 | 0 |
| 87 | 0.396 | 53 | 0.003 | 0.013 | 49 | 0.929 | 0.385 | 0.487 | 0.128 | 0.001 | 0.07 | 0.374 | 0.551 | 0.005 |
| 93 | 0.317 | 85 | 0.003 | -0.135 | 70 | 0.266 | 0.671 | 0.325 | 0.004 | 0 | 0.248 | 0.703 | 0.05 | 0 |
| 94 | 0.344 | 28 | 0.036 | 0.29 | 61 | 0.012 | 0.084 | 0.304 | 0.579 | 0.033 | 0.012 | 0.114 | 0.79 | 0.084 |
| 97 | 0.378 | 75 | 0.001 | -0.037 | 1488 | 0.154 | 1 | 0 | 0 | 0 | 1 | 0 | 0 | 0 |
| 106 | 0.382 | 36 | 0.021 | -0.223 | 47 | 0.133 | 0.777 | 0.22 | 0.003 | 0 | 0.497 | 0.469 | 0.034 | 0 |
| 107 | 0.222 | 86 | 0.039 | -0.105 | 158 | 0.19 | 0.939 | 0.061 | 0 | 0 | 0.722 | 0.278 | 0 | 0 |
| 110 | 0.554 | 280 | 0 | 0.091 | 144 | 0.278 | 0 | 0 | 0.176 | 0.824 | 0 | 0 | 0.176 | 0.824 |
| 111 | 0.333 | 57 | 0.011 | 0.226 | 118 | 0.014 | 0.041 | 0.369 | 0.59 | 0.001 | 0.003 | 0.1 | 0.897 | 0.001 |
| 112 | 0.701 | 11 | 0.014 | 0.75 | 11 | 0.006 | 0.031 | 0.066 | 0.245 | 0.658 | 0.003 | 0.012 | 0.129 | 0.856 |
| 113 | 0.681 | 126 | 0 | 0.764 | 177 | 0 | 0 | 0 | 0 | 1 | 0 | 0 | 0 | 1 |
| 114 | 0.571 | 32 | 0 | 0.653 | 32 | 0 | 0 | 0 | 0.04 | 0.96 | 0 | 0 | 0.019 | 0.981 |
| 115 | 0.502 | 33 | 0.003 | -0.45 | 10 | 0.199 | 0.362 | 0.355 | 0.228 | 0.055 | 0.052 | 0.161 | 0.518 | 0.269 |
| 116 | 0.288 | 174 | 0 | 0.323 | 141 | 0 | 0 | 0.003 | 0.996 | 0 | 0 | 0.001 | 0.999 | 0 |
| 118 | 0.214 | 113 | 0.023 | -0.049 | 160 | 0.54 | 0.846 | 0.154 | 0 | 0 | 0.43 | 0.569 | 0 | 0 |
| 120 | 0.38 | 31 | 0.017 | 0.25 | 43 | 0.053 | 0.154 | 0.354 | 0.452 | 0.039 | 0.025 | 0.15 | 0.715 | 0.111 |
| 122 | 0.722 | 9 | 0.026 | 0.923 | 18 | 0 | 0 | 0 | 0.017 | 0.983 | 0 | 0 | 0.007 | 0.993 |
| 124 | 0.384 | 36 | 0.02 | -0.034 | 70 | 0.779 | 0.639 | 0.352 | 0.01 | 0 | 0.324 | 0.594 | 0.083 | 0 |
| 127 | 0.685 | 30 | 0 | 0.527 | 27 | 0.004 | 0 | 0.001 | 0.06 | 0.939 | 0 | 0 | 0.028 | 0.972 |
| 133 | 0.446 | 25 | 0.025 | 0.423 | 39 | 0.007 | 0.026 | 0.104 | 0.525 | 0.346 | 0.002 | 0.017 | 0.381 | 0.601 |
| 134 | 0.21 | 117 | 0.023 | 0.5 | 236 | 0 | 0 | 0 | 0.33 | 0.67 | 0 | 0 | 0.31 | 0.69 |
| 136 | 0.498 | 30 | 0.004 | 0.102 | 58 | 0.446 | 0.288 | 0.492 | 0.217 | 0.003 | 0.058 | 0.296 | 0.629 | 0.018 |
| 145 | 0.769 | 78 | 0 | 0.653 | 38 | 0 | 0 | 0 | 0 | 1 | 0 | 0 | 0 | 1 |
| 146 | 0.65 | 16 | 0.005 | 0.497 | 13 | 0.085 | 0.063 | 0.122 | 0.323 | 0.492 | 0.005 | 0.021 | 0.2 | 0.775 |
| 148 | 0.189 | 196 | 0.008 | -0.03 | 261 | 0.628 | 0.857 | 0.143 | 0 | 0 | 0.344 | 0.656 | 0 | 0 |
| 149 | 0.189 | 196 | 0.008 | 0.018 | 316 | 0.746 | 0.671 | 0.329 | 0 | 0 | 0.151 | 0.849 | 0 | 0 |
| 150 | 0.723 | 15 | 0.002 | 0.208 | 20 | 0.385 | 0.13 | 0.227 | 0.39 | 0.252 | 0.016 | 0.058 | 0.35 | 0.576 |
| 151 | 0.4 | 43 | 0.007 | 0.003 | 126 | 0.975 | 0.596 | 0.402 | 0.003 | 0 | 0.279 | 0.697 | 0.024 | 0 |
| 153 | 0.86 | 9 | 0.002 | 0.12 | 9 | 0.768 | 0.18 | 0.229 | 0.304 | 0.287 | 0.036 | 0.078 | 0.275 | 0.611 |
| 154 | 0.43 | 70 | 0 | 0.11 | 16 | 0.69 | 0.036 | 0.12 | 0.588 | 0.256 | 0.001 | 0.022 | 0.609 | 0.368 |
| 155 | 0.31 | 53 | 0.023 | -0.034 | 72 | 0.778 | 0.657 | 0.337 | 0.006 | 0 | 0.294 | 0.638 | 0.068 | 0 |
| 158 | 0.375 | 40 | 0.017 | 0.411 | 95 | 0 | 0 | 0.01 | 0.686 | 0.305 | 0 | 0.001 | 0.535 | 0.464 |
| 161 | 0.483 | 46 | 0.001 | 0.178 | 46 | 0.239 | 0.066 | 0.236 | 0.625 | 0.073 | 0.004 | 0.054 | 0.779 | 0.163 |
| 167 | 0.595 | 19 | 0.006 | 0.254 | 23 | 0.245 | 0.144 | 0.256 | 0.408 | 0.192 | 0.016 | 0.067 | 0.42 | 0.498 |
| Mean | | | | | | | 0.293 | 0.234 | 0.217 | 0.256 | 0.126 | 0.285 | 0.267 | 0.321 |
